# 

**Published:** 1916-06

**Authors:** 


Yearly Vol. 71 June 1916 Number 11

### Buffalo Medical Journal

Established 1845 by AUSTIN FLINT, M. D.

EDITOR AND PUBLISHER:

A. L. BENEDICT, A.M., M.D. 228 Summer Street, Buffalo, N. Y.

ASSOCIATE EDITORS:

New York State:

Grover W. Wende, M. D., Buffalo.

C. W. Hennington, B.S., M.D., Rochester

Charles Haase, M. D., Elmira.

Willis E. Ford, M. D., Utica.

Martin B. Tinker, B. S., M. D., Ithaca.

H. I. Davenport, A. M., M. D., Auburn.

Foreign

Sir William Osler, M. D., LL. D., Oxford, Eng.

Prof. P. K. Pel, M. D., Amsterdam, Holland.

Rene Gaultier, M. D., Paris, France.

J. George Adami, M. D., LL.D., F. R. S. Montreal, Can.

Henry W. Hill, LL. D., Buffalo, Associate Editor in Medical Jurisprudence.

